# An evolutionary preserved intergenic spacer in gadiform mitogenomes generates a long noncoding RNA

**DOI:** 10.1186/s12862-014-0182-3

**Published:** 2014-08-22

**Authors:** Tor Erik Jørgensen, Ingrid Bakke, Anita Ursvik, Morten Andreassen, Truls Moum, Steinar D Johansen

**Affiliations:** 1 Marine Genomics group, Faculty of Biosciences and Aquaculture, University of Nordland, Bodø, Norway; 2 Department of Biotechnology, Norwegian University of Science and Technology, Trondheim, Norway; 3 Department of Medical Biology, Faculty of Health Sciences, UiT – Norwegian Arctic University, MH-building Breivika, Tromsø, N-9037, Norway

**Keywords:** Atlantic cod, European hake, Heteroplasmy, lncRNA, Mitogenome

## Abstract

**Background:**

Vertebrate mitogenomes are economically organized and usually lack intergenic sequences other than the control region. Intergenic spacers located between the tRNA^Thr^ and tRNA^Pro^ genes (“T-P spacers”) have been observed in several taxa, including gadiform species, but information about their biological roles and putative functions is still lacking.

**Results:**

Sequence characterization of the complete European hake *Merluccius merluccius* mitogenome identified a complex T-P spacer ranging in size from 223–532 bp. Further analyses of 32 gadiform species, representing 8 families and 28 genera, revealed the evolutionary preserved presence of T-P spacers across all taxa. Molecular complexity of the T-P spacers was found to be coherent with the phylogenetic relationships, supporting a common ancestral origin and gain of function during codfish evolution. Intraspecific variation of T-P spacer sequences was assessed in 225 Atlantic cod specimens and revealed 26 haplotypes. Pyrosequencing data representing the mito-transcriptome poly (A) fraction in Atlantic cod identified an abundant H-strand specific long noncoding RNA of about 375 nt. The T-P spacer corresponded to the 5’ part of this transcript, which terminated within the control region in a tail-to-tail configuration with the L-strand specific transcript (the 7S RNA).

**Conclusions:**

The T-P spacer is inferred to be evolutionary preserved in gadiform mitogenomes due to gain of function through a long noncoding RNA. We suggest that the T-P spacer adds stability to the H-strand specific long noncoding RNA by forming stable hairpin structures and additional protein binding sites.

## Background

One characteristic feature of vertebrate mitochondrial genomes (mitogenomes) is the economical gene organization. Vertebrates usually lack mitochondrial intergenic sequences of any appreciable size other than the approximately 1-kb control region (CR) and the 30-bp origin of light (L) strand replication (OriL). The CR harbors the origin of heavy (H) strand replication (OriH) and initiation sites for H-strand and L-strand transcription, as well as the displacement loop (D-loop), but does not contain any canonical protein coding or RNA coding genes [1].

Among the 2600 completely sequenced vertebrate mitogenomes, a great majority shares an identical arrangement of 37 genes. However, alternative gene orders due to minor rearrangements and sequence duplications have been noted [2],[3]. A common feature of most rearrangements is DNA duplications that involve tRNA genes and intergenic sequences. Mitogenomes of several snake species contain two CR copies [4],[5], and the four gene orders known in birds differ in CR copy number and CR location [6]-[8]. Related rearrangements have also been noted in bony fishes, and include both CR, OriL and tRNA gene duplications [9]-[12].

A mitogenome intergenic spacer between the tRNA^Thr^ and tRNA^Pro^ genes (the T-P spacer), located close to the CR, has been reported in a few species representing distantly related vertebrate taxa. Short T-P spacers have been found in ostrich birds [13], in some toad linages including *Xenopus*[14],[15], and in the mole lizard [16]. Larger T-P spacers have been reported in various species of the salamander family Ambystomatidae [17],[18]. These complex spacers vary in size from 240 bp to 680 bp and tandem repeat sequence motifs appear common, but no functional role has yet been assigned. A T-P spacer in a fish species was first discovered in the Atlantic cod (*Gadus morhua*) mitogenome [19]-[21], and later noted in the related Walleye pollock (*Theragra chalcogramma*) [22] and six additional gadiform species representing three families (Gadidae, Lotidae and Phycidae) [23]. These T-P spacers vary in size from 25 bp in fourbeard rockling (*Enchelyopus cimbrius*) to 99 bp in haddock (*Melanogrammus aeglefinus*), and contain one or two copies of a conserved 17-bp motif (Box-motif) [23]. T-P spacers demonstrate interspecific sequence variation and have been applied as genetic markers in population studies of both Walleye pollock [22] and Atlantic cod [24]. More recently T-P spacers were reported in two additional gadid species [25]. While Greenland cod (*Gadus ogac*) contains two size variants of the spacer (73 bp and 102 bp), highly similar in sequence to that of Atlantic cod, the Arctic cod (*Arctogadus glacialis*) T-P spacer appears more complex and variable in size due to short heteroplasmic tandem repeat motifs [25].

The vertebrate mitochondrial transcriptome (mito-transcriptome) has been investigated in human cells and tissues [26],[27]. The H and L strand polycistronic precursor transcripts are processed into the 22 tRNAs, 2 rRNAs, and 11 mRNAs [28]. Here, the L-strand specific promoter (LSP) generates a transcript that gives rise to the ND6 mRNA and 8 tRNAs. The H-strand specific transcription, however, is more complex since two promoters (HSP_1_ and HSP_2_) are involved. Whereas the HSP_1_-specific transcript is short and highly abundant, and is the main source of mitochondrial rRNAs, the HSP_2_-specific transcript is processed into 8 monocistronic and two bicistronic mRNAs [26],[28]. This organization appears highly conserved among vertebrates since Atlantic cod and saithe (*Pollachius virens*) mito-transcriptomes are very similar to that of humans [29].

Long noncoding RNAs (lncRNAs), typically longer than 200 nt, are highly abundant in vertebrates where they possess key roles in gene regulation linked to tissue specificity, development and disease [30],[31]. The human mitogenome also code for lncRNAs in addition to their 37 canonical genes. The first mitochondrial lncRNA to be discovered was the 7S RNA, transcribed from LSP within the CR [32],[33]. Additional mitochondrial lncRNAs have since then been identified in human cells, and include lncND5, lncND6, lncCytB [27], as well as the prognostic LIPCAR lncRNAs antisense to CytB and COII mRNAs [34]. The lncND5 RNA is of particular interest due to its antisense organization to ND5 mRNA, and the fact that ND5 is the only tightly regulated protein gene in vertebrate mitochondria [35],[36]. Mitochondrial lncRNAs appear highly conserved among vertebrates, and we recently reported noncoding RNA corresponding to lncND5 in the mito-transcriptomes of Atlantic cod and saithe [29].

Here we report two non-overlapping mitochondrial lncRNAs (lncCR-H and lncCR-L) transcribed from opposite strands within the Atlantic cod CR, both terminating at the termination-associated sequence (TAS). While the 500 nt lncCR-L RNA apparently corresponds to the human 7S RNA [29], the 375 nt lncCR-H RNA has not been reported previously. The 5’ end of lncCR-H corresponds to the T-P spacer that apparently adds stability to the noncoding RNA. The T-P spacer was found to be present in all 32 gadiform species investigated (representing 8 families and 28 genera), and feature analysis indicates directional evolution of spacers from a simple organization in early branching gadiform families (e.g. Moridae or Macrouridae) to complex structures including heteroplasmy in Merluciidae and Gadidae.

## Results

### Complete mitogenome sequence of the European hake

**Figure 1:**
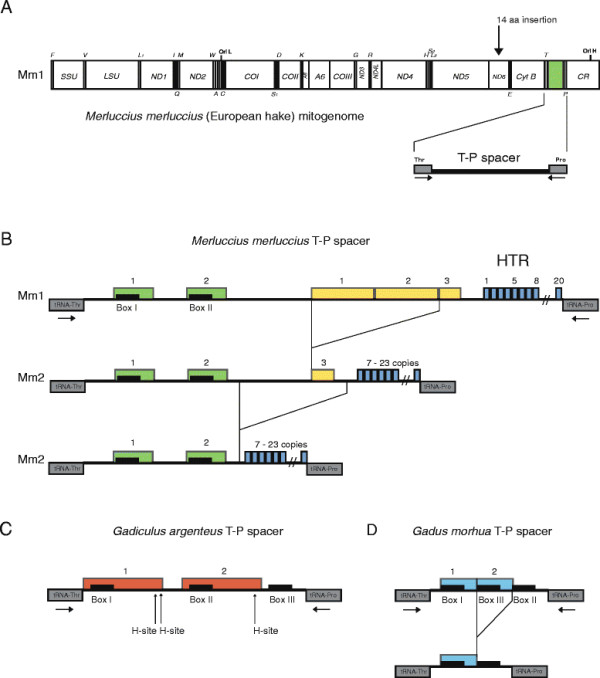
**Organization of T-P spacer. A**. Gene content and organization of European hake mitochondrial genome (Mm1 specimen) presented as a linear map of the circular mtDNA. All protein genes, except ND6, are encoded by the H-strand. Abbreviations: SSU and LSU, mitochondrial small- and large-subunit ribosomal RNA genes; ND1-6, NADH dehydrogenase subunit 1 to 6; COI-III, cytochrome c oxidase subunit I to III; A6 and A8, ATPase subunit 6 and 8; CytB, cytochrome B; oriH and oriL, origin of H-strand and L-strand replication; CR, control region containing the D-loop; tRNA genes are indicated by the standard one-letter symbols for amino acids. H-strand and L-strand encoded tRNA genes are indicated above and below the diagram, respectively. T-P spacer is indicated below the diagram, and position of the 42-bp insertion in the ND6 gene corresponding to 14 amino acids is indicated by arrow above. **B**. Organization of the European hake T-P spacer in specimens Mm1 and Mm2. Different direct repeat motifs are indicated by green (conserved repeats), yellow (optional repeats), and blue (heteroplasmic tandem repeats, HTR) boxes. Two copies of the Box motif are present. **C**. Organization of the Silvery pout (*G. argenteus*) T-P spacer containing three Box-motifs. The third copy of the direct repeat (red boxes) is truncated. Heteroplasmic sites (H) are indicated. **D**. Organization of the Atlantic cod (*G. morhua*) T-P spacer. About 1% (3/225) of analyzed specimens harbor a direct repeat duplication (blue box).

The 17.078 bp mitogenome of European hake (*Merluccius merluccius*) was determined and represents the first complete mitogenome sequence in the family Merlucciidae (Order Gadiformes). The circular mitogenome contains the same set of two ribosomal RNA genes, 13 protein coding genes, and 22 transfer RNA genes as reported in all codfishes to date (Figure 1A), and is arranged according to the general vertebrate organization [1]. The 871 bp CR was found to be similar in size and related in sequence to that of the southern hake *M. australis* (Additional file 1: Figure S1). We noted an unusual 42 bp insertion in the ND6 gene, corresponding to 14 amino acids, in European hake compared to that of other gadiforms (Additional file 2: Figure S2). The insertion is located in a non-transmembrane region known to contain insertions in mammals [37]. Finally, a large and complex intergenic T-P spacer (532 bp) was found between the tRNA^Thr^ and tRNA^Pro^ genes (Figure 1A).

The phylogenetic position of European hake among Gadiformes was assessed from concatenated mitochondrial protein sequences (3814 amino acid positions) derived from complete mitogenome sequences (Additional file 3: Table S1), and a representative neighbor-joining tree is presented in Additional file 4: Figure S3. Eight gadiform families were included in the analysis and European hake (family Merlucciidae) was found to occupy a more basal position compared to Gadidae and Lotidae, but apparenty more recent than Macrouridae, Bregmacerotidae and Moridae. These observations are in general agreement with previous works on gadiform phylogeny [38]-[40].

### T-P spacer in European hake contains heteroplasmic tandem repeats

The T-P spacer included in the complete European hake mitogenome sequence (Mm1) was determined to 532 bp. This sequence consists of a 34 bp conserved direct repeat containing the 17-bp Box-motif [23], an optional direct repeat of about 60 bp, and 20 copies of an 8-bp heteroplasmic tandem repeat (HTR) (Figure 1B; Additional file 5: Figure S4A). To investigate individual sequence variations, a T-P spacer region from another specimen (Mm2) was analyzed by PCR amplification, plasmid cloning, and subsequent DNA sequencing. The Mm2 T-P spacer showed extensive length heteroplasmy, varying in size from 258 bp to 430 bp (Figure 1B; Additional file 5: Figure S4B). Size variation was partly caused by the 8-bp HTR motif, which varied from 7 to 23 copies among six sequenced clones. Furthermore, four clones contained an additional 76 bp sequence (Figure 1B). All plasmid clones investigated gave identical sequences, except the HTR copy number and two single nucleotide polymorphic sites (Additional file 5: Figure S4B).

### Distribution and variability of T-P spacers among gadiform species

To address the phylogenetic distribution of T-P spacers among gadiform species, we determined the DNA sequences for the corresponding region in 9 additional species (Table 1). We also retrieved available sequences from the NCBI database (Table 1, Additional file 6: Figure S5), adding up to a total of 32 species representing eight families (Gadidae, Lotidae, Ranicipitidae, Merlucciidae, Phycidae, Macrouridae, Moridae and Bregmacerotidae). Several interesting features were noted: (1) all of the gadiform species investigated harbor mitochondrial T-P spacers. (2) Extensive size variation was observed among species, from 7 bp in Anadara (*Bathygadus antrodes*) to 532 bp in European hake. (3) A conserved 17 bp sequence (Box-motif) in 1 to 3 copies was present in 6 of 8 gadiform families. (4) Heteroplasmic features were observed in three species, namely Arctic cod [25], European hake, and silvery pout (*Gadiculus argenteus*), a codfish species belonging to the family Gadidae. In a similar approach to that of the European hake, the silvery pout spacer was PCR amplified, cloned and sequenced. Four independent clones revealed small length variations of homopolymeric stretches and one heteroplasmic SNP site (Additional file 5: Figure S4C). The T-P spacer was found to be 261–263 bp in size, carrying three copies of the Box-motif (Figure 1C).

**Table 1:** Key features of gadiform T-P spacers

**Species**	**Common name**	^**1**^**Size (bp)**	^**2**^**Box**	^**3**^**Reference**
**Order: Gadiformes**				
**Family: Gadidae**				
*Gadus morhua*	(Atlantic cod)	74/103	2/3	AM489716; [22],[23]
*Gadus ogac*	(Greenland cod)	73/102	2/3	FJ396453; [24]
*Theragra chalcogramma*	(Alaska Pollock)	70-72	2	Y17984; [22]
*Theragra finnmarchica*	(Norwegian Pollock)	72	2	AM489718
*Boreogadus saida*	(Polar cod)	70	2	Y17985; [22]
*Arctogadus glacialis*	(Arctic cod)	*291	2	[24]
*Melanogrammus aeglefinus*	(Haddock)	99	2	Y17986; [22]
*Merlangius merlangius*	(Whiting)	69	2	DQ020496
*Pollachius virens*	(Saithe)	59	1	FR751399
*Pollachius pollachius*	(Pollack)	50	1	FR751400
*Trisopterus esmarkii*	(Norwegian pout)	77	1	This work
*Trisopterus minutes*	(Poor cod)	75	1	This work
*Micromesistius poutassou*	(Blue whiting)	48	1	FR751401; [22]
*Gadiculus argenteus*	(Silvery pout)	*261-263	3	This work
**Family: Lotidae**				
*Lota lota*	(Burbot)	103	1	AP004412
*Brosme brosme*	(Tusk)	48	1	Y17988; [22]
*Molva molva*	(Ling)	83	1	This work
*Molva dipterygia*	(Blue ling)	59	1	This work
**Family: Phycidae**				
*Enchelyopus cimbrius*	(Fourbeaed rockling)	25	1	Y17989; [22]
*Gaidropsarus argentatus*	(Tree-bearded rockling)	48	1	Y17990; [22]
*Phycis blennoides*	(Greater forkbeard)	28	1	This work
**Family: Ranicipitidae**				
Raniceps raninus	(Tadpole fish)	39	1	This work
**Family: Merlucciidae**				
*Macruronus novaezelandiae*	(Hoki)	59	1	This work
*Merluccius merluccius*	(European hake)	*223-532	2	FR751402; This work
**Family: Macrouridae**				
*Squalogadus modificatus*	(Tadpole whiptail)	47	1	AP008989
*Trachyrincus murragi*	(Roughnose grenadier)	64	1	AP008990
*Bathygadus antrodes*	(Anadara)	7	-	AP008988
*Caelorinchus kishinouyei*	(Mugara grenadier)	12	-	AP002929
*Coryphaenoides rupestris*	(Roundnose grenadier)	21	-	This work
*Ventrifossa garmani*	(Sagami grenadier)	72	-	AP008991
**Family: Bregmacerotidae**				
*Bregmaceros nectabanus*	(Smallscale codlet)	(+)	-	AP004411
**Family: Moridae**				
*Physiculus japonicus*	(Japanese codling)	16	-	AP004409
*Laemonema longipes*	(Longfin codling)	33	-	AB108839
**Order: Lophiiformes**				
**Family: Lophiidae**				
*Lophius piscatorius*	(Frogfish)	0	-	This work
*Lophius americanus*	(American angler)	0	-	AP00414

Notes: ^1^Sequence and size variants of T-P spacers are given in Additional file 6: Figure S5. T-P spacers of *M. merluccius*, *G. argenteus*, and *A. glacialis* are heteroplasmic (*). The T-P spacer of *B. nectabanus* (+) is involved in gene order rearrangements. ^2^Box-motif copy number. ^3^Data base accession number and key references.

### Intraspecific variation of the Atlantic cod T-P spacer

**Figure 2:**
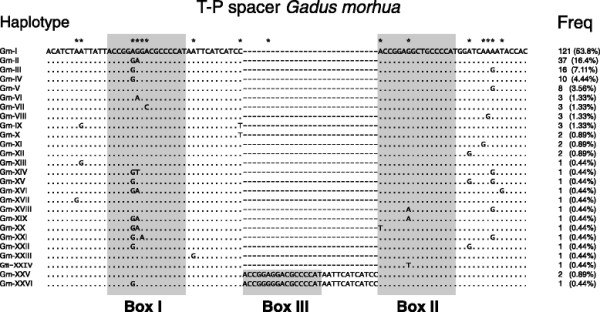
**Intraspecific variation of Atlantic cod T-P spacer.** Alignment of 225 specimens revealed 26 distinct haplotypes. Dots indicate identical positions to the reference Gm-I haplotype and dashes indicate deletions. Two haplotypes (Gm-XXV and Gm-XXVI) harbor a 29 bp insertion. The conserved 17-bp motif is boxed, and the 16 variable sites are indicated by stars above the Gm-I haplotype sequence. Right; numbers of specimens and frequency (%) belonging to each haplotype.

Intraspecific variation of the T-P spacer was investigated in Atlantic cod. The T-P spacer of Atlantic cod is normally 74 bp in size and contains two copies of the Box-motif (Figure 1D). A total of 225 specimens were included in the analysis, representing all main localities in the North Atlantic Ocean [41]. Among these, 115 sequences were published previously [19],[24],[42],[43] and 110 new sequences were obtained by PCR amplifications and sequencing. We found variable sites at 16 of the 74 positions, which defined 26 T-P spacer haplotypes (Figure 2). The Box-motif II appeared more conserved than motif I, but no heteroplasmic sites were detected. The dominant haplotype (Gm-I) was represented by 53.8% (121 of 225 individuals), including the whole genome sequenced NEAC_001 specimen [44]. These findings are in general agreement with intraspecific variation previously reported in the T-P spacer of 110 individuals of Walleye pollock [23]. Three individuals representing two haplotypes (Gm-XXV and Gm-XXVI) contained a 29 bp insert that includes the 17-bp Box-motif copy (Figure 1D). A similar duplication of Box-motif I was observed in Greenland cod [25].

### Analysis of mito-transcriptome data from Atlantic cod liver tissue

**Figure 3:**
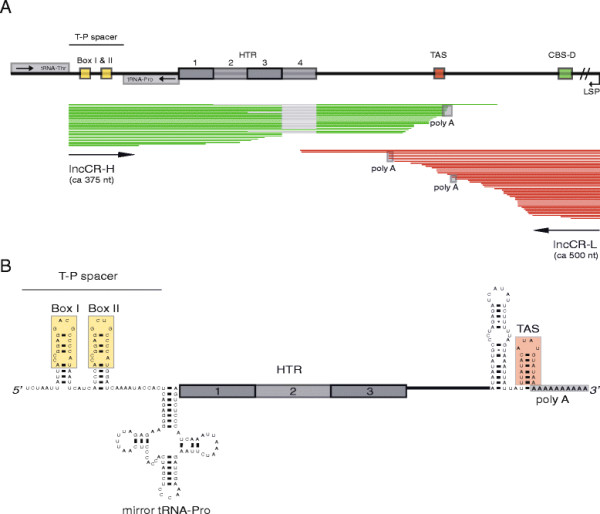
**Long noncoding RNAs generated from CR and T-P spacer. A**. 454 read coverage of the Atlantic cod liver mitochondrial transcriptome at the T-P spacer and control region. H-strand specific reads (green) start exactly 3 nt downstream the first position of the T-P spacer, and represent lncCR-H. L-strand specific reads (red) represent lncCR-L. Grey line in lncCR-H indicates missing HTR motif due to HTR copy number variation. **B**. Secondary structure diagram of lncCR-H in Atlantic cod. The T-P spacer is located at the 5’ end of the RNA, and Box-motifs are indicated (yellow boxes). The mirror tRNA^Pro^ structure and TAS hairpin structure (red box) are located upstream and downstream, respectively, of the heteroplasmic tandem repeat (HTR) motifs.

We reported previously the mapping of 4698 pyrosequencing reads of mitochondrial transcripts from Atlantic cod liver tissue [29]. Here, all mitochondrial mRNAs as well as the ribosomal RNA transcript were represented by multiple reads each. We also observed a significant and stable L-strand specific lncRNA (lncCR-L) of about 500 nt covering parts of the CR, and apparently similar to that of the 7S RNA in human mitochondria [29]. A closer inspection of the mito-transcriptome data identified a number of reads mapping to the opposite part of the CR including the T-P spacer (Figure 3A), and corresponding to a H-strand specific lncRNA. Interestingly, this approximately 375 nt transcript (lncCR-H) starts exactly 3 nt upstream of the tRNA^Thr^ gene and includes the T-P spacer, proceeds into the mirror tRNA^Pro^ and further into the HTR domain of the CR, and terminates by a non-template polyA tail immediately downstream of the TAS. Based on read map coverage, the relative abundance of lncCR-H was comparable to that of mitochondrial mRNAs (e.g. ND4L mRNA [29]). LncCR-H varies in size due to different numbers of HTRs included in the RNA. We observed 1–4 HTR copies in lncCR-H, with a majority of 3 copies (Figure 3A). A secondary structure diagram of lncCR-H is presented in Figure 3B, indicating structured domains at both the 5’ and 3’ ends. Interestingly, the Box and TAS motifs are proposed to be part of tetraloop hairpin structures.

## Discussion

We report a complex intergenic spacer between the tRNA^Thr^ and tRNA^Pro^ genes in the European hake mitochondrial genome. The T-P spacer contains HTRs, and significant size variation was observed among specimens. Furthermore, we found T-P spacers to be present in mitogenomes of all the 32 gadiform species investigated, representing 8 families and 28 genera, and the majority of spacers harbor a conserved 17-bp Box-motif. Analysis of the Atlantic cod liver mito-transcriptome identified a long noncoding RNA (lncCR-H) that contains the T-P spacer within its 5’ end.

**Figure 4:**
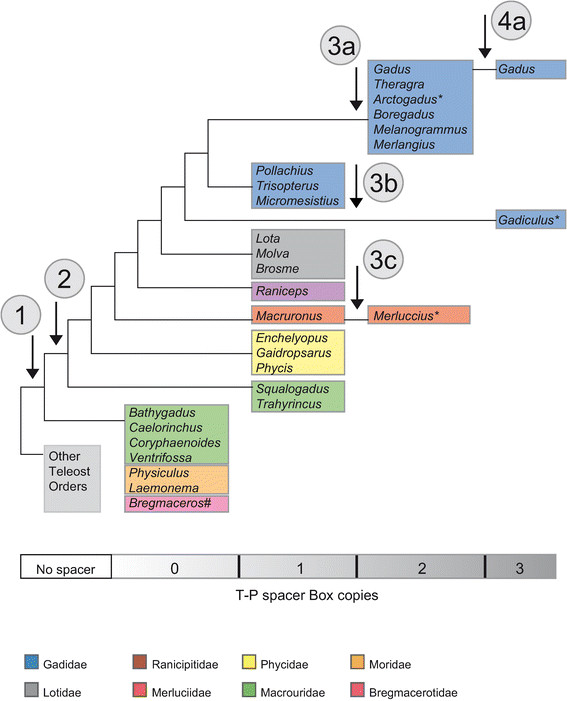
**Evolutionary history of gadiform T-P spacer.** Key events in the evolutionary history of the T-P spacer are indicated by numbers 1–4 and explained in the main text. In short: 1, first occurrence of spacer; 2, first occurrence of Box-motif; 3abc, duplication of Box-motif; 4, second duplication of Box motif. Genus containing T-P spacer with heteroplasmic feature is indicated by star (*). The *Bregmaceros* spacer is involved in gene order rearrangements (#). The 32 gadiform species represent 20 genera and 8 families (color-coded). Pattern of relationship between gadiform families is extrapolated from [38]–[40].

Mitogenomes of most bony fishes conform to the general vertebrate gene organization, and lack intergenic nucleotides between the tRNA^Thr^ and tRNA^Pro^ genes. In contrast, we found the presence of intergenic spacers at this location to be a consistent feature among gadiform species. Based on key features and the distribution pattern of gadiform T-P spacers (Table 1), as well as the current understanding of gadiform phylogeny [38]-[40], we propose the following evolutionary scenario for the gadiform T-P spacer (Figure 4). This scenario implies gain of function for this sequence element during gadiform evolution. (1) A spacer sequence was introduced at a basal point of gadiform phylogenesis. (2) Once established, the Box-motif became a preserved sequence feature of descendant taxa after the split from Bathygadus, Caelorinchus, Coryphaenoides, Ventrifossa, Physiculus, Laemonema, and Bregmaceros. The majority of gadiform genera, representing 6 families, contain a single copy of the Box-motif. (3a) Duplication of the Box-motif then occurred within the family Gadidae, leaving six genera (*Gadus*, *Theragra*, *Arctogadus*, *Boregadus*, *Melanogrammus* and *Merlangius*) with two consecutive Box-motifs. (3b) The Box-motif was also independently duplicated in silvery pout (*Gadiculus*), and (3c) within the family Merluciidae, resulting in the complex European hake T-P spacer. (4) A second duplication occurred recently in *Gadus*. Here, a subset of Atlantic cod and Greenland cod specimens contains three copies of the Box-motif due to a duplication of Box-motif I. T-P spacer heteroplasmy was only noted in genera with duplicated Box-motifs (*Merluccius*, *Gadiculus* and *Arctogadus*).

Higher order phylogenetic relationships within Gadiformes are still weakly supported by molecular data [38]-[40]. Other branching patterns than those depicted here would imply instances of secondary loss of the Box-motif, which can of course not be precluded. However, the Box-motif apparently represents a selective feature, of which the exact functional role is still unknown. It is interesting to note that complex T-P spacers in salamanders [17],[18] contains strikingly similar Box-motifs to those reported in codfishes (Additional file 7: Table S2), suggesting that an evolutionary conserved mitochondrial factor may interact with the motif.

Analysis of the Atlantic cod liver mito-transcriptome identified a relatively abundant lncRNA (lncCR-H) of about 375 nt, that contains the T-P spacer sequence within its 5’ end. The first nucleotide of lncCR-H corresponds to 3 nt downstream the tRNA^Thr^ gene, and the 5’ end of the lncRNA was probably generated after RNase Z processing of the tRNA precursor [45]. The 3’ end of lncCR-H appears heterogenous and polyadenylated, and corresponds to sequences in proximity to the TAS box element within the CR. LncCR-H harbours secondary structure elements, including a mirror tRNA^Pro^ and tetraloop helices, probably adding stability to the RNA. Interestingly, recent RNA-Seq experiments in breast cell lines identified an expressed human homolog to lncCR-H (our unpublished results), and HeLa cell RNA profiles supported the existence of this mitochondrial lncRNA (see Figure 1B in [27]). Thus, lncCR-H appears conserved at a wide taxonomic range, which implies a functional role in vertebrate mitochondria.

Mitochondrial lncRNAs corresponding to antisense transcripts of mRNAs have been characterized in human cells [26],[27],[34] as well as in Atlantic cod [29]. In that study we identified a second mitochondrial lncRNA within CR, named lncCR-L. The L-strand specific lncCR-L, which corresponds to the human 7S RNA [32],[33], is apparently initiated at LSP and terminated at TAS. Our finding implicates that TAS is a transcription termination site both for H-strand and L-strand specific lncRNAs. TAS in Atlantic cod consists of a perfect inverted repeat motif (*UUAUACAU*AUGUAUAA) and thus forms identical tetraloop hairpin structures at the H- and L-strand transcripts. TAS-binding proteins have been reported in human, rat and bovine mitochondria [46],[47]. How lncCR-H and lncCR-L are terminated and regulated in Atlantic cod, as well as their biological functions, are currently unknown. However, we speculate that the biological role of the T-P spacer is to add stability to an evolutionary conserved lncRNA where the Box-motif forms an RNA structure signal to be recognized by mitochondrial proteins. lncCR-H may have a role in antisense regulation by premature termination of L-strand transcription at TAS, resulting in a coordination of H and L transcript levels. Anyway, these possibilities need further investigations in different cell and tissue types, and in different species.

## Conclusions

Two long noncoding RNA candidates from the mitochondrial control region were described in gadiform fishes. These RNAs were transcribed from different DNA strands, and found to terminate at the TAS region in a tail-to-tail configuration. Whereas the L-strand specific lncRNA (lncCR-L) corresponded to the previously described mitochondrial 7S RNA, lncCR-H was found to be unique by containing a 5’ domain of an intergenic spacer. Analyses of 32 gadiform species revealed that a T-P spacer was present in all investigated species, representing 8 families and 28 genera. Some species contained complex spacer sequences due to conserved motifs, duplications, and heteroplasmy. We propose that the T-P spacer adds stability to lncCR-H by forming stable hairpin structures and additional protein binding sites, but a biological role of lncCR-H is currently unknown.

## Methods

### DNA extraction and PCR amplification

DNA was extracted from muscle tissue samples as previously described [11],[38],[42],[48]. DNA for complete mitogenome sequencing of European hake was extracted from frozen muscle tissue by using the mtDNA Extractor CT Kit from Wako [42]. The PCR and sequencing primers (Additional file 8: Table S2) applied in the European hake sequencing were designed from the published Atlantic cod mitogenome sequence [21] and other available gadiform sequences (Additional file 3: Table S1). These primers were used to amplify the mitogenomes in 1 to 4 kb fragments essentially as described by [11]. The primer pair L15498/H15666 was used for amplification of the T-P spacer from most species. For European hake, a specific primer set (L15498/H15642) was designed for T-P spacer amplifications. The PCR products were treated with exonuclease and shrimp alkaline phosphatase (USB) prior to plasmid cloning and sequencing. The amplified fragments were subcloned into the *Sma*I-site of pUC18 vector using the Sure-Clone Ligation kit (Pharmacia Biotech).

### DNA sequencing

Sanger sequencing of the European hake mitogenome and Atlantic cod T-P spacers was performed directly on PCR products as previously described [11] using the BigDye kit (Applied Biosystems). Sequencing primers are found in Additional file 8: Table S2. Manual sequencing reactions were performed on most T-P spacers using the Thermo Sequencing Terminator Cycle Sequencing kit (Amersham) and [α-^33^P] ddNTP (Amersham) as the label. Sequencing reactions were run on 7 M urea/6% polyacrylamide gels after denaturation at 85°C for 2 minutes. Roche 454 pyrosequencing of the Atlantic cod transcriptome was performed as a service given by Eurofins MWG Operon (Germany). A poly(A) enriched normalized liver-specific cDNA library (based on total cellular RNA) was generated by random hexamer first strand synthesis. About 1.2 million reads were obtained from pooling equal amounts of total RNA from 10 Atlantic cod specimens [29].

### Data analyses

A sequence alignment representing complete mitogenomes of 20 gadiform species and one out-group was generated using T-Coffee v/9 [49] with manual refinements. The alignment was build from the complete set of protein codons (except stop codons) creating a concatenated sequence of 11442 nt positions (3814 codons) corresponding to the 13 protein genes. The tree-building methods of neighbor-joining (NJ), maximum parsimony (MP) and maximum likelihood (ML) in MEGA version 5 [50] were used to reconstruct molecular phylogenies. NJ trees were build using Jones-Taylor-Thornton model. MP trees were reconstructed using the Close-Neighbor-Interchange search option. ML trees were built from best-fit models of protein evolution generated by MEGA 5 and ProtTest 3 [51]. The topologies of the ME, MP and ML trees were evaluated by bootstrap analyses (2000 replications). Analyses of mito-transcriptome 454 pyrosequencing reads were as previously described [29].

## Competing interests

The authors declare that they have no competing interests.

## Authors’ contributions

TEJ and IB participated in DNA sequencing and design of the study, performed analysis, and contributed in the discussions of the results. AU and MA participated in mitogenome sequencing and transcriptome analysis, respectively. TM participated in the design of this study and in discussions of results. SDJ directed the research, participating in the analysis, and wrote the manuscript in collaboration with all the authors. All authors read and approved the final manuscript.

## Additional files

## Supplementary Material

Additional file 1: Figure S1. DNA sequence alignment of hake mitochondrial control region. Dots indicate identical positions to the European hake reference sequence and dashes indicate deletions. Conserved sequence elements (red letters) recognized among gadiform mitochondrial CRs [21] are indicated. TAS, termination-associated sequence; Py-RUN, pyrimidine-rich segment; CSB 2, 3, and D, conserved sequence blocks. European hake – *Merluccius merluccius* (Mmer), FR751402; Southern hake – *M. australis* (Maus), FJ423612. Click here for file

Additional file 2: Figure S2. Amino acid sequence alignment of gadiform ND6 protein. The 14 amino acid insertion between trans-membrane domains (TMD) IV and V is indicated by red letters. The TMD annotations are according to [37]. Click here for file

Additional file 3: Table S1. Complete gadiform mitogenomes. Click here for file

Additional file 4: Figure S3. Gadiform phylogeny based on mitogenome-derived amino acid sequences. Neighbor joining (NJ) phylogenetic tree based on the alignment of 13 concatenated proteins corresponding to 3814 amino acids. Bootstrap values (%) from 2000 replicates, all over 40%, are shown at branches. The values are from NJ, maximum parsimony (MP) and maximum likelihood (ML) analyses. Red filled circles indicate highly significant branch points of bootstrap values of 100% in the NJ, MP and ML tree construction methods. Different families are color-coded. References to the complete mitogenome sequences are found in Additional file 3: Table S1. Note that Macrouridae appears paraphyletic. Click here for file

Additional file 5: Figure S4. European hake and Silvery pout T-P spacer sequence variants. A) DNA sequence of the European hake T-P spacer in specimen Mm1 (FR751402). Different direct repreat motifs are indicated. DR-a (green), conserved direct repeat containing the 17-bp Box-motif sequence; DR-b (yellow), optional direct repeat, including a truncated (*) copy; HTR (blue), heteroplasmic tandem repeat. B) Long heteroplasmic variant in the Mm2 specimen. Two single nucleotide positions (red) found to be heteroplasmic are indicated. C) Short heteroplasmic variant in the Mm2 specimen. D) Silvery pout T-P spacer including heteroplasmic direct repeats (DR) boxed in red. Click here for file

Additional file 6: Figure S5. Gadiform T-P spacer sequences. Box-motif is underlined. The different gadiform families are shown in A to G. Click here for file

Additional file 7: Table S2. Box-motif sequence compilation. Click here for file

Additional file 8: Table S3. PCR and sequencing primer specifications. Click here for file

## References

[B1] Wallace DC Why do we still have a maternally inherited mitochondrial DNA? Insights from evolutionary medicine Annu Rev Biochem 2007 76 781 821 17506638 10.1146/annurev.biochem.76.081205.150955

[B2] Brown C Dowling TE Brown WM Evolution of animal mitochondrial DNA: relevance for population biology and systematics Annu Rev Ecol Syst 1987 18 269 292

[B3] San Mauro D Gower DJ Zardoya R Wilkinson M A hotspot of gene order rearrangement by tandem duplication and random loss in the vertebrate mitochondrial genome Mol Biol Evol 2006 23 227 234 16177229 10.1093/molbev/msj025

[B4] Kumazawa Y Ota H Nishida M Ozawa T Gene rearrangements in snake mitochondrial genomes: highly concerted evolution of control-region-like sequences duplicated and inserted into a tRNA gene cluster Mol Biol Evol 1996 13 1242 1254 8896377 10.1093/oxfordjournals.molbev.a025690

[B5] Kumazawa Y Ota H Nishida M Ozawa T The complete nucleotide sequence of a snake (*Dinodon semicarinatus*) mitochondrial genome with two identical control regions Genetics 1998 150 313 329 9725849 10.1093/genetics/150.1.313 PMC1460336

[B6] Mindell DP Sorenson MD Dimcheff DE Multiple independent origins of mitochondrial gene order in birds Proc Natl Acad Sci U S A 1998 95 10693 10697 9724766 10.1073/pnas.95.18.10693 PMC27957

[B7] Eberhard JR Wright TF Bermingham E Duplication and concerted evolution of the mitochondrial control region in the parrot genus *Amazona* Mol Biol Evol 2001 18 1330 1342 11420371 10.1093/oxfordjournals.molbev.a003917

[B8] Abbott CL Double MC Trueman JW Robinson A Cockburn A An unusual source of apparent mitochondrial heteroplasmy: duplicate mitochondrial control regions in *Thalassarche albatrosses* Mol Ecol 2005 14 3605 3613 16156827 10.1111/j.1365-294X.2005.02672.x

[B9] Inoue JG Miya M Tsukamoto K Nishida M Complete mitochondrial DNA sequence of *Conger myriaster* (Teleostei: Anguilliformes): novel gene order for vertebrate mitochondrial genomes and the phylogenetic implications for Auguilliform families J Mol Evol 2001 52 311 320 11343127 10.1007/s002390010161

[B10] Satoh TP Miya M Endo H Nishida M Round and pointed-head grenadier fishes (Actinopterygii: Gadiformes) represent a single sister group: evidence from complete mitochondrial genome sequences Mol Phylogen Evol 2006 40 129 138 10.1016/j.ympev.2006.02.014 16603389

[B11] Breines R Ursvik A Nymark M Johansen SD Coucheron DH Complete mitochondrial genome sequences of the Arctic Ocean codfishes *Arctogadus glacialis* and *Boreogadus saida* reveal oriL and tRNA gene duplications Polar Biol 2008 31 1245 1252

[B12] Poulsen JY Byrkjedal I Willassen E Rees D Takeshima H Satoh TP Shinohara G Nishida M Miya M Mitogenomic sequences and evidence from unique gene rearrangements corroborate evolutionary relationships of myctophiformes (Neoteleostei) BMC Evol Biol 2013 13 111 23731841 10.1186/1471-2148-13-111 PMC3682873

[B13] Härlid A Janke A Arnason U The mtDNA sequence of the ostrich and the divergence between Paleognathous and Neognathous birds Mol Biol Evol 1997 14 754 761 9214748 10.1093/oxfordjournals.molbev.a025815

[B14] Roe BA Ma DP Wilson RK Wong JF The complete nucleotide sequence of the *Xenopus laevis* mitochondrial genome J Biol Chem 1985 260 9759 9774 4019494

[B15] Lloyd RE Foster PG Guille M Littlewood DTJ Next generation sequencing and comparative analyses of *Xenopus* mitogenomes BMC Genomics 2012 13 496 22992290 10.1186/1471-2164-13-496 PMC3546946

[B16] Macay JR Schulte JA Larson A Papenfuss TJ Tandem duplications via light-strand synthesis may provide a precursor for mitochondrial genomic rearrangement Mol Biol Evol 1998 15 71 75 9491606 10.1093/oxfordjournals.molbev.a025849

[B17] McKnight ML Shaffer HB Large, rapidly evolving intergenic spacers in the mitochondrial DNA of the salamander family Ambystomatidae (Amphibia: Caudata) Mol Biol Evol 1997 14 1167 1176 9364774 10.1093/oxfordjournals.molbev.a025726

[B18] Samuels AK Weisrock DW Smith JJ France KJ Walker JA Putta S Voss SR Transcriptional and phylogenetic analysis of five complete ambystomatid salamander mitochondrial genomes Gene 2005 349 43 53 15780978 10.1016/j.gene.2004.12.037

[B19] Johansen S Guddal PH Johansen T Organization of the mitochondrial genome of Atlantic cod, *Gadus morhua* Nucleic Acids Res 1990 18 411 419 2308841 10.1093/nar/18.3.411 PMC333442

[B20] Johansen S Johansen T Sequence analysis of 12 structural genes and a novel non-coding region from mitochondrial DNA of Atlantic cod, *Gadus morhua* Biochem Biophys Acta 1994 1218 213 217 8018725 10.1016/0167-4781(94)90015-9

[B21] Johansen S Bakke I The complete mitochondrial DNA sequence of Atlantic cod (*Gadus morhua*): relevance to taxonomic studies among codfishes Mol Mar Biol Biotechnol 1996 5 203 214 8817926

[B22] Shields GF Gust JR Lack of geographic structure in mitochondrial DNA sequences of Bering Sea walleye Pollock, *Theragra chalcogramma* Mol Mar Biol Biotechnol 1995 4 69 82 7749468

[B23] Bakke I Shields GF Johansen S Sequence characterization of a unique intergenic spacer in gadiformes mitochondrial DNA Mar Biotechnol 1999 1 411 415 10525675 10.1007/pl00011797

[B24] Sigurgíslason H Árnason E Extent of mitochondrial DNA sequence variation in Atlantic cod from the Faroe Islands: a resolution of gene genealogy Heredity 2003 91 557 564 14560303 10.1038/sj.hdy.6800361

[B25] Pálsson S Paulsen J Árnason E Rapid evolution of the intergenic T-P spacer in the mtDNA of Arctic cod *Arctogadus glacialis* Mar Biotechnol 2008 10 270 277 18214612 10.1007/s10126-007-9058-5

[B26] Mercer TR Neph S Dinger ME Crawford J Smith MA Shearwood AMJ Haugen E Bracken CP Rackham O Stamatoyannopoulos JA Filipovska A Mattick JS The human mitochondrial transcriptome Cell 2011 146 645 658 21854988 10.1016/j.cell.2011.06.051 PMC3160626

[B27] Rackham O Shearwood AMJ Mercer TR Davies SMK Mattick JS Filipovska A Long noncoding RNAs are generated from the mitochondrial genome and regulated by nuclear-encoded proteins RNA 2011 17 2085 2093 22028365 10.1261/rna.029405.111 PMC3222122

[B28] Temperley RJ Wydro M Lightowlers RN Chrzanowska-Lightowlers ZM Human mitochondrial mRNAs – like members of all families, similar but different Biochim Biophys Acta 2010 1797 1081 1085 20211597 10.1016/j.bbabio.2010.02.036 PMC3003153

[B29] Coucheron DH Nymark M Breines R Karlsen BO Andreassen M Jørgensen TE Moum T Johansen SD Characterization of mitochondrial mRNAs in codfish reveals unique features compared to mammals Curr Genet 2011 57 213 222 21484258 10.1007/s00294-011-0338-2 PMC3097352

[B30] Fatica A Bozzoni I Long non-coding RNAs: new players in cell differentiation and development Nature Rev Genet 2014 15 7 21 24296535 10.1038/nrg3606

[B31] Necsulea A Soumillon M Warnefors M Liechti A Daish T Zeller U Baker JC Grutzner F Kaessmann H The evolution of lncRNA repertoires and expression patterns in tetrapods Nature 2014 505 635 640 24463510 10.1038/nature12943

[B32] Ojala D Crews S Montoya J Gelfand R Attardi G A small polyadenylated RNA (7S RNA), containing a putative ribosome attachment site, maps near the origin of human mitochondrial DNA replication J Mol Biol 1981 150 303 314 6172590 10.1016/0022-2836(81)90454-x

[B33] Chang DD Clayton DA Precise identification of individual promoter for transcription of each strand of human mitochondrial DNA Cell 1984 36 635 643 6697390 10.1016/0092-8674(84)90343-x

[B34] Kumarswamy R Bauters C Volkmann I Maury F Fetisch J Holzmann A Lemesle G de Groote P Pinet F Thum T Circulating long noncoding RNA, LIPCAR, predicts survival in patients with heart failure Circ Res 2014 114 1569 1575 24663402 10.1161/CIRCRESAHA.114.303915

[B35] Bai Y Shakeley RM Attardi G Tight control of respiration by NADH dehydrogenase ND5 subunit gene expression in mouse mitochondria Mol Cell Biol 2000 20 805 815 10629037 10.1128/mcb.20.3.805-815.2000 PMC85197

[B36] Chomyn A Mitochondrial genetic control of assembly and function of complex I in mammalian cells J Bioenerg Biomembr 2001 33 251 257 11695835 10.1023/a:1010791204961

[B37] Moum T Willassen NP Johansen S Intragenic rearrangements in the mitochondrial NADH dehydrogenase subunit 6 gene of vertebrates Curr Genet 1994 25 554 557 8082208 10.1007/BF00351677

[B38] Bakke I Johansen SD Molecular phylogenetics of gadidae and related gadiformes based on mitochondrial DNA sequences Mar Biotechnol 2005 7 61 69 15759085 10.1007/s10126-004-3131-0

[B39] Teletchea F Laudet V Hanni C Phylogeny of the Gadidae (sensu Svetovidov, 1948) based on their morphology and two mitochondrial genes Mol Phylogen Evol 2006 38 189 199 10.1016/j.ympev.2005.09.001 16311046

[B40] Roa-Varón A Ortí G Phylogenetic relationships among families of Gadiformes (Teleostei, Paracanthopterygii) based on nuclear and mitochondrial data Mol Phylogenet Evol 2009 52 688 704 19345274 10.1016/j.ympev.2009.03.020

[B41] Johansen SD Coucheron DH Andreassen M Karlsen BO Furmanek T Jørgensen TE Emblem Å Breines R Nordeide JT Moum T Nederbragt AJ Stenseth NC Jakobsen KS Large-scale sequence analyses of Atlantic cod N Biotechnol 2009 25 263 271 19491044 10.1016/j.nbt.2009.03.014

[B42] Ursvik A Breines R Christiansen JS Fevolden S-E Coucheron DH Johansen SD A mitogenomic approach to the taxonomy of pollocks: *Theragra chalcogramma* and *T. finnmarchica* represent one single species BMC Evol Biol 2007 7 87 17555567 10.1186/1471-2148-7-86 PMC1894972

[B43] Karlsen BO Emblem A Jørgensen TE Klingan KA Nordeide JT Moum T Johansen SD Mitogenome sequence variation in migratory and stationary ecotypes of North-east Atlantic cod Mar Genomics 2014 15 103 108 24456931 10.1016/j.margen.2014.01.001

[B44] Star B Nederbragt AJ Jentoft S Grimholt U Malmstrøm M Gregers TF Rounge TB Paulsen J Solbakken MH Sharma A Wetten OF Lanzén A Winer R Knight J Vogel JH Aken B Andersen O Lagesen K Tooming-Klunderud A Edvardsen RB Tina KG Espelund M Nepal C Previti C Karlsen BO Moum T Skage M Berg PR Gjøen T Kuhl H The genome sequence of Atlantic cod reveals a unique immune system Nature 2011 477 207 210 21832995 10.1038/nature10342 PMC3537168

[B45] Rossmanith W Of P and Z: Mitochondrial tRNA processing enzymes Biochim Biophys Acta 2012 1810 1017 1026 22137969 10.1016/j.bbagrm.2011.11.003 PMC3790967

[B46] Madsen CS Ghivizzani SC Hauswirth WW Protein binding to a single termination-associated sequence in the mitochondrial DNA D-loop region Mol Cell Biol 1993 13 2162 2171 8455604 10.1128/mcb.13.4.2162-2171.1993 PMC359537

[B47] Roberti M Musicco C Polosa PL Milella F Gadaleta MN Cantatore P Multiple protein-binding sites in the TAS-region of human and rat mitochondrial DNA Biochem Biophys Res Com 1998 243 36 40 9473475 10.1006/bbrc.1997.8052

[B48] Bakke I Johansen S Characterization of mitochondrial ribosomal RNA genes in gadiformes: sequence variations, secondary structural features, and phylogenetic implications Mol Phylogen Evol 2002 25 87 100 10.1016/s1055-7903(02)00220-8 12383753

[B49] Notredame C Higgins DG Heringa J T-Coffee: A novel method for fast and accurate multiple sequence alignment J Mol Biol 2001 302 205 217 10964570 10.1006/jmbi.2000.4042

[B50] Tamura K Peterson D Peterson N Stecher G Nei M Kumar S MEGA5: Molecular Evolutionary Genetics Analysis Using Maximum Likelihood, Evolutionary Distance, and Maximum Parsimony Methods Mol Biol Evol 2011 28 2731 2739 21546353 10.1093/molbev/msr121 PMC3203626

[B51] Abascal F Zardoya R Posada D ProtTest: selection of best-fit models of protein evolution Bioinform 2005 21 2104 2105 10.1093/bioinformatics/bti263 15647292

