# Supplementary material for: An evolutionary preserved intergenic spacer in gadiform mitogenomes generates a long noncoding RNA
Source: BMC Evol Biol. 2014 Aug 22;14:182. doi: 10.1186/s12862-014-0182-3 (PMC4236577; doi:10.1186/s12862-014-0182-3)
Supplement: Additional file 1: Figure S1. — DNA sequence alignment of hake mitochondrial control region. Dots indicate identical positions to the European hake reference sequence and dashes indicate deletions. Conserved sequence elements (red letters) recognized among gadiform mitochondrial CRs [21] are indicated. TAS, termination-associated sequence; Py-RUN, pyrimidine-rich segment; CSB 2, 3, and D, conserved sequence blocks. European hake – Merluccius merluccius (Mmer), FR751402; Southern hake – M. australis (Maus), FJ423612. [file s12862-014-0182-3-S1.pdf]

Additional file 1: Figure S1

```
                                               TAS
Mmer    ACGGCACTTCCCCTGCAATGTAAGACTTGAACATATATGTATTATCCCCATTCTCCTATATTAACCATTCAGGCAATTT
Maus    ..A....AC.....A.............T.....G...............GT.TT...T.A.........T.......

Mmer    AAAATTGAAAAAAGAACATTAACATAAAATTAAACTTACCATTACTCGTATTTAACCTGTCAACCCATAATACTCATTC
Maus    ...T...............AG...C..T..A.T.TC..T........CC.CCC..T..A..............A.....

Mmer    TTTCGTACA-TTAAATTTATTTGTATCCCCTCATTTCCTTTCCCCAAATACCCTAATTAAATCACCCGCTAGACTTGCT
Maus    ....C....A.................T.................T..G......................A.......

                                                                              CSB-D
Mmer    TACTAAGATACACGTTTCAGTGTAAGGTCAAGGGTACTACTCGAAGACTCACCACTCAGTGAATTATTCCTGGCATCCC
Maus    ...C..A......AG....A...................A.T.....T.....G..........................

Mmer    TGCCTAGCTTCAGGTCCATAAACGTCTAACAGCTCACAACTTGCATTTTTGTCCATCTCTTATTGTTCGCAGTCATATA
Maus    ...................G.GA...T.T.A....A....A.........CA.........A....G...........

                                                               Py-RUN
Mmer    TTCAAATTTCTCAGCATGCCGAGCGTTCTCTCTAAGGGGCAACGGGTTTTCTTTTTTTT--TTCCTTTCATCTGGCATC
Maus    ..........C.........G....................A......T........CT.........C.......T

Mmer    CCAGAGTGAACACGGTAGTTC-TCGTCAGGGTTGAACTTGCTCTTGGTATGAAGAAAATTTATATCATGTTAGAAGGGC
Maus    TT.C.T..C.........C..C.G....A...........................G.....GAT......A......

Mmer    TTAAAGTAAGAGTAGCATATAGATATATCATGAGCATAATATGAGAAATTTTCTCGAAGGTTTTCTATTGCGCCCCCTT
Maus    .G..G....A...........AG...T....................T...G........A........ATA.......

                                                    CSB-2
Mmer    CTTTAATT-ACGCGATTTTTTGCGTAGGCCCCCCCTACCCCCCCAATTCTCCTGAGATGTCTATAACTCTGTCGGCCCC
Maus    ........T....A...............................T.....G.....-....GG.T..........

        CSB-3
Mmer    CCCGGGAACAGAAGACCCTCGAGAACTGAGAAAAAAAGATAACTCTATTTT---AAAAAATTATCTAGTTTTTAATTAT
Maus    ......G......A..TT.G.G.G.T........G..-...-..T......TTAG..CG.C...T........TT.A.C

Mmer    CACTCTACCCCTTGTTTTGTATTCAA-TAGCAAT-ATATTCTTAAAATGTGTTTTGTTTATTATAGTATTACAATATTA
Maus    ....T.-...GGG....AA.......G..A....C.A...................A.....C...............

Mmer    CAATAT----------------TGATT-T
Maus    ......TACAATACTTGTAATG.....A.
```
