# Supplementary material for: An evolutionary preserved intergenic spacer in gadiform mitogenomes generates a long noncoding RNA
Source: BMC Evol Biol. 2014 Aug 22;14:182. doi: 10.1186/s12862-014-0182-3 (PMC4236577; doi:10.1186/s12862-014-0182-3)
Supplement: Additional file 2: Figure S2. — Amino acid sequence alignment of gadiform ND6 protein. The 14 amino acid insertion between trans-membrane domains (TMD) IV and V is indicated by red letters. The TMD annotations are according to [37]. [file s12862-014-0182-3-S2.pdf]

Additional file 2: Figure S2

```
                TMD I                TMD II               TMD III
Mmer   MAYLMLSFLVGMIVGVISVASNPSPYFGALGLVLMAGAGCGCLIGHGGAFLAVVLFLIYLGGMLVVFAYCAALAAEPYPEAWGDVEVF
Smod   MTYVMLTLLIGVIFGVISVASNPSPYFAALGLVLLAGVGCGILIGHGGSFLSLVLFLIYLGGMLVVFAYCAALAAEPYPEAWGEWSIL
Tmur   MAYMMLILLVGVIFGVISVASNPSPYFAALGLVLLAGVGCGILMGHGGSFLSLVLFLIYLGGMLVVFAYCAALAAEPYPEAWGEWAIM
Bnec   MSYVLILILIGMILGVVSVVSSPSPYFAALGLVVMAAAGCSVLVVSGGSFLSLVLFLIYLGGMLVVFAYSAALAAEPYPEAWGEWSVL
Pjap   MMYL-LVVLAVMNLGVIFVASNPTPNFASLGLVFAAGLGCVVVLGSGGSFLALVLFLIYLGGMLVVFAYCVALAAEEYPEGLGAWSVS
Ckis   MFYMMMMMMVGFVLGAAALASNPSPVYAVLGLVLMAGMACMILINSGGPFLSLLFFLVYLGGMLVVFAHCVALAAERYPKAWGDVIFL
Vgar   MSYSMMMLSGVFILGAVALASSPSPVYAALGLVLMAGGASMLLIDSGGPFLSLLFYLVYLGGVLVVFAHCVALAADNYPKAWGDVILL
Llot   MAYIMLTLLIGMVLGVISVASNPSPYFAALGLVLVAGVGCGVLMGHGGSFLSLVLFLIYLGGMLVVFAYCAALAAEPYPEAWGEWSVL
Mpou   MAYIMLTLLVGMVLGVISVASNPSPYFAALGLVLVAGVGCVILMGHGGSFLSLVLFLIYLGGMLVVFAYCAALAAEPYPEAWGEWSVL
Pvir   MAYIMLTLLIGMVLGVISVASNPSPYFAALGLVLVAGVGCVVLMGHGGSFLSLVLFLIYLGGMLVVFAYCAALAAEPYPEAWGEWSVL
Ppol   MAYIMLTLLVGMVLGVISVASNPSPYFAALGLVLVAGVGCVVLMGHGGSFLSLVLFLIYLGGMLVVFAYCAALAAEPYPEAWGEWSVL
Mmea   MAYIMLTLLVGMVLGVISVASNPSPYFAALGLVLVAGVGCVVLMGHGGSFLSLVLFLIYLGGMLVVFAYCAALAAEPYPEAWGEWSVL
Maeg   MAYIMLTLLIGMVLGVISVASNPSPYFAALGLVLVAGVGCVVLMGHGGSFLSLVLFLIYLGGMLVVFAYCAALAAEPYPEAWGEWSVL
Tfin   MAYIMLTLLIGMVLGVISVASNPSPYFAALGLVLVAGVGCVVLMGHGGSFLSLVLFLIYLGGMLVVFAYCAALAAEPYPEAWGEWSVL
Tcha   MAYIMLTLLIGMVLGVISVASNPSPYFAALGLVLVAGVGCVVLMGHGGSFLSLVLFLIYLGGMLVVFAYCAALAAEPYPEAWGEWSVL
Goga   MAYIMLTLLIGMVLGVISVASNPSPYFAALGLVLVAGVGCVVLMGHGGSFLSLVLFLIYLGGMLVVFAYCAALAAEPYPEAWGEWSVL
Gmor   MAYIMLTLLIGMVLGVISVASNPSPYFAALGLVLVAGVGCVVLMGHGGSFLSLVLFLIYLGGMLVVFAYCAALAAEPYPEAWGEWSVL
Gmac   MAYIMLTLLIGMVLGVISVASNPSPYFAALGLVLVAGVGCVVLMGHGGSFLSLVLFLIYLGGMLVVFAYCAALAAEPYPEAWGEWSVL
Mpro   MAYIMLTLLVGMVLGVISVASNPSPYFAALGLVLVAGVGCVVLMGHGGSFLSLVLFLIYLGGMLVVFAYCAALAAEPYPEAWGEWSVL
Bsai   MAYIMLTLLIGMVLGVISVASNPSPYFAALGLVLVAGVGCVVLMGHGGSFLSLVLFLIYLGGMLVVFAYCAALAAEPYPEAWGEWSVL
Agla   MAYIMLTLLIGMVLGVISVASNPSPYFAALGLVLVAGVGCVVLMGHGGSFLSLVLFLIYLGGMLVVFAYCAALAAEPYPEAWGEWSVL
Ulom   MTYFVLFLGLCFVLGGLAVASNPSPYYGVVGLVLASVAGCAWLLSLGVSFVSLVLFMVYLGGMLVVFVYSVSLAADPFPEAWGDWRVV
Hsap   MMYALFLLSVGLVMGFVGFSSKPSPIYGGLVLIVSGVVGCVIILNFGGGYMGLMVFLIYLGGMMVVFGYTTAMAIEEYPEAWGSGVEV
```

```
                 TMD IV           Insert                          TMD V
Mmer   AAALFYFLLVFGGAFWFFGGGYGAGWASVEEVVAFPAIFGDPFDEVINFSVISGDAAGVGMLYSLGGGLLVLSAWVLLLTLFVVLEVT
Smod   GSVLGYFSLVLGALFWFVGGWHEGAWV--------------PADELIEFSVVSGDSAGVAMMYSLGGGMLIIGAWVLLLALFVVLELA
Tmur   GYALGYLLSVLGALSWFWGGWYEGGWV--------------PVNELVEFSVVPGDSGGVALMYSSGGGFLVVGAWVLLLALFVVLELA
Bnec   GAVGGYLVVIMGAVIWFYNSLSEGEWL--------------TSEDTDGKGVFGLDSEGVALMYSSGGGLLILSAWVLLLTLFVVLSVT
Pjap   SVIAGYVALLAGGTVFFMEEWYQYIWV--------------PESKSSDYGLIIPDVGGAGVMYHQGAGMLLIGAFVLLLTLFVVLEIC
Ckis   GALVGVVVGYSVAVIVGWVNWFGVGSE--------------SGYDFDDYSFICPEGEGLGLLYDKGGWLLLFCVIILLVVLVVVLELT
Vgar   MVLVGITVSWTAAAVFNWLDWSSLALL--------------NTYDYGDFTLLYPEGEGLSLMYDDGGWFLFFCVYVLLVVLALVLEVT
Llot   GSVLGYLLLVFGGVSWFWGGWYEGAWV--------------PVDELIEFSVVAADSGGVALMYSLGGGLLVVSAWVLLLTLLVVLELT
Mpou   GSVLGYLLLVVGAGSWFWEGWYEAMWV--------------PVDELIEFSVVAADSGGVALMYSLGGGLLVASAWVLLLTLLVVLELT
Pvir   GSVLGYLLLVLGAGSWFWGGWYEGMWV--------------PVDELIEFSVVAADSGGVALMYSLGGGLLVVSAWVLLLTLLVVLELT
Ppol   GSVLGYLLLVLGAGSWFWGGWYEGMWV--------------PVDELIEFSVVAADSGGVALMYSLGGGLLVVSAWVLLLTLLVVLELT
Mmea   GAVLGYLLLVVGAGSWFWGGWYEGMWV--------------PVDELIEFSVVEADSGGVALMYSLGGGLLVVSAWVLLLTLLVVLELT
Maeg   GSAMGYLLLVVGAGSWFWGGWYEGMWV--------------PVDELIEFSVVAADLGGVALMYSLGGGLLVVSAWVLLLTLLVVLELT
Tfin   GSVLGYLLLVVGAGSWFWGGWYEGMWV--------------PVDELIEFSVVAADSGGVALMYSLGGGLLVVSAWVLLLTLLVVLELT
Tcha   GSVLGYLLLVVGAGSWFWGGWYEGMWV--------------PVDELIEFSVVAADSGGVALMYSLGGGLLVVSAWVLLLTLLVVLELT
Goga   GSVLGYLLLVVGAGSWFWGGWYEGMWV--------------PVDELIEFSVVAADSGGVALMYSLGGGLLVVSAWVLLLTLLVVLELT
Gmor   GSVLGYLLLVVGAGSWFWGGWYEGMWV--------------PVDELIEFSVVAADSGGVALMYSLGGGLLVVSAWVLLLTLLVVLELT
Gmac   GSVLGYLLLVVGAGSWFWGGWYEGMWV--------------PVDELIEFSVVAADSGGVALMYSLGGGLLVVSAWVLLLTLLVVLELT
Mpro   GSVLGYLLLVLGAGSWFWGGWYEGMWV--------------PVDELIEFSVTAADSGGVALMYSLGGGLLVVSAWVLLLTLLVVLELT
Bsai   GSVLGYLLLVLGAGSWFWGGWYEGMWV--------------PVDELIEFSVVAADSGGVALMYSLGGGLLVVSAWVLLLTLLVVLELT
Agla   GSVLGYLLLVVGAGSWFWGGWYEGMWV--------------PVDELIEFSVVAADSGGVALMYSLGGGLLVASAWVLLLTLLVVLELT
Ulom   GYGMGFVAVLVMGMVVGGFECWDLGVV--------------TVDSVGMFSV-RLDFGGVAMFYSCGVGMFLVAGWGLLLTLFVVLELV
Hsap   LVSVLVGLAMEVGLVLWVKEYDGVVVV-----VNFNSVGSWMIYEGEGSGLIREDPIGAGALYDYGRWLVVVTGWTLFVGVYIVIEIA
```

Mmer	RGLARGALRAV		*Merluccius merluccius* (European hake); FR751402
Smod	RGRAWGTMRAV		*Squalogadus modificatus* (Tadpole whiptail); AP008989
Tmur	RGRAWGTLRAV		*Trachyrincus murragi* (Roughnose grenadier); AP008990
Bnec	WGRAEGSLRVV		*Bregmaceros nectabanus* (Smallscale codlet); AP004411
Pjap	RGLSRGTLRSV		*Physiculus japonicas* (Japanese codling); AP004409
Ckis	RGRSQGALRAI		*Caelorinchus kishinouyei* (Mugara grenadier); AP002929
Vgar	RGGSYGALRAI		*Ventrifossa garmani* (Sagami grenadier); AP008991
Llot	RGLARGALRAV		*Lota lota* (Burbot); AP004412
Mpou	RGLARGALRAV		*Micromesistius poutassou* (Blue whiting); FR751401
Pvir	RGLARGALRAV		*Pollachius virens* (Saithe); FR751399
Ppol	RGLARGALRAV		*Pollachius pollachius* (Pollack); FR751400
Mmea	RGLARGALRAV		*Merlangius merlangius* (Whiting); DQ020496
Maeg	RGLARGALRAV		*Melanogrammus aeglefinus* (Haddock); AM489717
Tfin	RGLARGALRAV		*Theragra finnmarchica* (Norwegian Pollock); AM489718
Tcha	RGLARGALRAV		*Theragra chalcogramma* (Alaska Pollock); AB094061
Goga	RGLARGALRAV		*Gadus ogac* (Greenland cod); DQ356941
Gmor	RGLARGALRAV		*Gadus morhua* (Atlantic cod); AM489716
Gmac	RGLARGALRAV		*Gadus macrocephalus* (Pacific cod); DQ356937
Mpro	RGLARGALRAV		Microgadus proximus (Pacific tomcod); DQ356944
Bsai	RGLARGALRAV		*Boreogadus saida* (Polar cod); AM919428
Agla	RGLARGALRAV		*Arctogadus glacialis* (Arctic cod); AM919429
Ulom	RGLTRGAIRAV	(bird)	Uria lomvia (thick-billed guillemot); X73914
Hsap	RGN--------	(human)	*Homo sapiens*; KC417443
