# Supplementary material for: An evolutionary preserved intergenic spacer in gadiform mitogenomes generates a long noncoding RNA
Source: BMC Evol Biol. 2014 Aug 22;14:182. doi: 10.1186/s12862-014-0182-3 (PMC4236577; doi:10.1186/s12862-014-0182-3)
Supplement: Additional file 3: Table S1. — Complete gadiform mitogenomes. [file s12862-014-0182-3-S3.pdf]

Additional file 3: Table S1

Complete gadiform mitogenomes

	Species	Common name	Acc #
<u>**Order: Gadiformes**</u>			
**Family: Gadidae**			
	Gadus morhua	(Atlantic cod)	AM489716
	Gadus ogac	(Greenland cod)	DQ489716
	Theragra chalcogramma	(Alaska Pollock)	AB094061
	Theragra finnmarchica	(Norwegian Pollock)	AM489718
	Boreogadus saida	(Polar cod)	AM919428
	Arctogadus glacialis	(Arctic cod)	AM919429
	Melanogrammus aeglefinus	(Haddock)	AM489717
	Merlangius merlangius	(Whiting)	DQ020496
	Pollachius virens	(Saithe)	FR751399
	Pollachius pollachius	(Pollack)	FR751400
	Micromesistius poutassou	(Blue whiting)	FR751401
**Family: Lotidae**			
	Lota lota	(Burbot)	AP004412
**Family: Merlucciidae**			
	Merluccius merluccius	(European hake)	FR751402
**Family: Macrouridae**			
	Bathygadus antrodes	(Anadara)	AP008988
	Coelorinchus kishinouyei	(Mugara grenadier)	AP002929
	Squalogadus modificatus	(Tadpole whiptail)	AP008989
	Trachyrincus murragi	(Roughnose grenadier)	AP008990
	Ventrifossa garmani	(Sagami grenadier)	AP008991
**Family: Bregmacerotidae**			
	Bregmaceros nectabanus	(Smallscale codlet)	AP004411
**Family: Moridae**			
	Physiculus japonicus	(Japanese codling)	AP004409
<u>**Order: Lophiiformes**</u>			
**Family: Lophiidae**			
	Lophius americanus	(American angler)	AP004414
