# Supplementary figures and images for: An evolutionary preserved intergenic spacer in gadiform mitogenomes generates a long noncoding RNA
Source: BMC Evol Biol. 2014 Aug 22;14:182. doi: 10.1186/s12862-014-0182-3 (PMC4236577; doi:10.1186/s12862-014-0182-3)

## Additional file 4: Figure S3

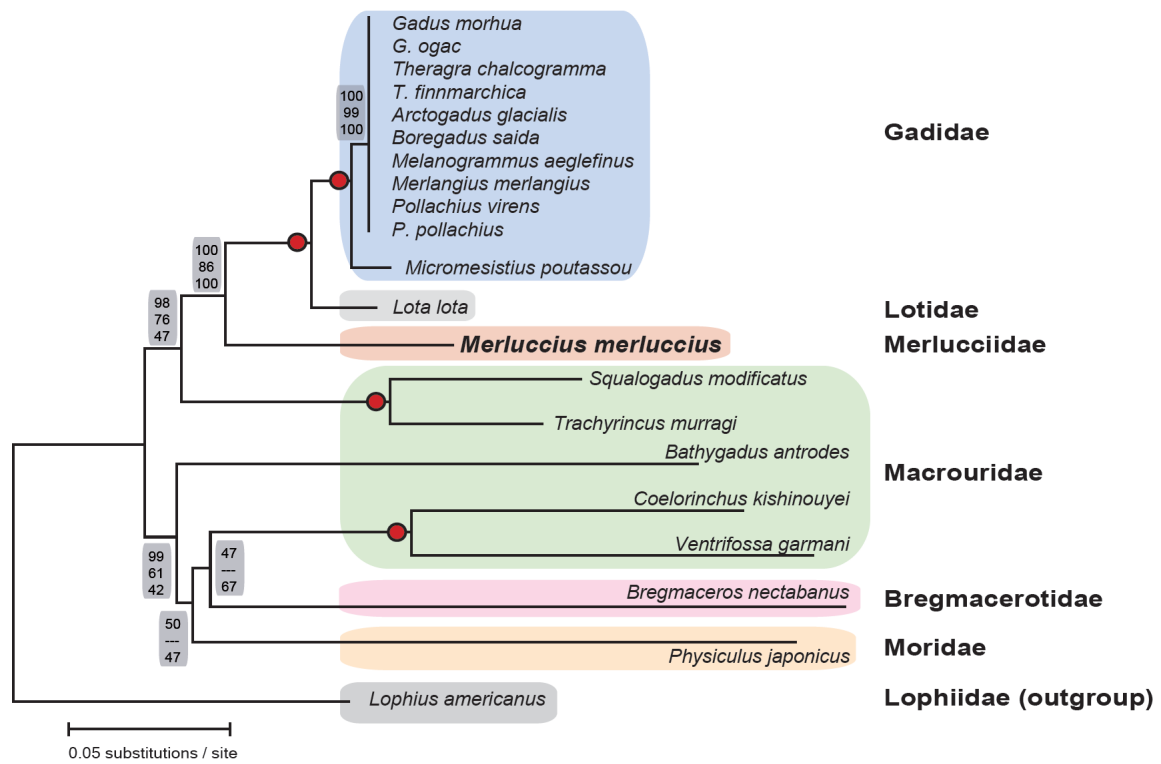

Supplement: Additional file 4: Figure S3. — Gadiform phylogeny based on mitogenome-derived amino acid sequences. Neighbor joining (NJ) phylogenetic tree based on the alignment of 13 concatenated proteins corresponding to 3814 amino acids. Bootstrap values (%) from 2000 replicates, all over 40%, are shown at branches. The values are from NJ, maximum parsimony (MP) and maximum likelihood (ML) analyses. Red filled circles indicate highly significant branch points of bootstrap values of 100% in the NJ, MP and ML tree construction methods. Different families are color-coded. References to the complete mitogenome sequences are found in Additional file 3: Table S1. Note that Macrouridae appears paraphyletic. [file s12862-014-0182-3-S4.pdf]
