# Supplementary material for: An evolutionary preserved intergenic spacer in gadiform mitogenomes generates a long noncoding RNA
Source: BMC Evol Biol. 2014 Aug 22;14:182. doi: 10.1186/s12862-014-0182-3 (PMC4236577; doi:10.1186/s12862-014-0182-3)
Supplement: Additional file 5: Figure S4. — European hake and Silvery pout T-P spacer sequence variants. A) DNA sequence of the European hake T-P spacer in specimen Mm1 (FR751402). Different direct repreat motifs are indicated. DR-a (green), conserved direct repeat containing the 17-bp Box-motif sequence; DR-b (yellow), optional direct repeat, including a truncated (*) copy; HTR (blue), heteroplasmic tandem repeat. B) Long heteroplasmic variant in the Mm2 specimen. Two single nucleotide positions (red) found to be heteroplasmic are indicated. C) Short heteroplasmic variant in the Mm2 specimen. D) Silvery pout T-P spacer including heteroplasmic direct repeats (DR) boxed in red. [file s12862-014-0182-3-S5.pdf]

Additional file 5: Figure S4

A) European hake (*M. merluccius*); Mm1

Box I DR-a1

AAACACGAGCAGCACAATTGCCTCCGAGCTCTGCCCAACCCACTTTCCTACAAAACCTTAAGAGTCTTAACCCCGCAGGTCCCAAACCCC

Box II DR-a2

GAGCTCTGCCCAACTACTTTTCCTGCAAAAGGTCCCGACTAAAAAATCCTACCACAGACCCCCCCCCTTTTTAAACCCGCCCTGCCCAGC

DR-b1

CCACTTTCGTCAGGAAGACCCGGACCCAAAAAAATCTTCATCTTTCCCCCCCCCCCTTTTTTTAACCCCCCCCCCCCCCCAAAGCCGGAC

DR-b2 DR-b3*

CCAAAAAAATCTTCATCTTTCCCCCCCCCCCTTTTTTTAACCCCCCCCCAAAGCCGGACCCAAAAAATCTTCATCCTTTTTTAACCCCCC

HTR (20 copies)

CCTTTTTTACCCGCTTACCCGCTTACCCGCTTACCCGCTTACCCGCTTACCCGCTTACCCGCTTACCCGCTTACCCGCTTACCCGCTTAC

CCGCTTACCCGCTTACCCGCTTACCCGCTT ACCCGCTTACCCGCTTACCCGCTTACCCGCTTACCCGCTTACCCGCTTACCC

B) European hake (*M. merluccius*); Mm2 - long

Box I DR-a1

AAACACGAGCAGCACAATTGCCTCCGAGCTCTGCCCAACCCACTTTCCTACAAAACCCTTAAGAGTCTTAACCCCGCAGGTCCCAAACCC

Box II DR-a2

CGAGCTCTGCCCAACTACTTTTCCTGCAAAAGGTCCCGACTAAAGAATCCTACCACAGACCCCCCCCCCCTTTTAAACCCGCCCTGCCCA

DR-b3* HTR (7-23 copies)

GCCCACTTTCGTCAGGAAGAGCCGGACCCAAAAAAATCTTCATCTTTCCCCCCCCCTTTTTTACCCGCTTACCCGCTTACCCGCTTACCC

GCTTACCCGCTTACCCGCTTACCCGCTTACCC

C) European hake (*M. merluccius*); Mm2 - short

Box I DR-a1

AAACACGAGCAGCACAATTGCCTCCGAGCTCTGCCCAACCCACTTTCCTACAAAACCCTTAAGAGTCTTAACCCCGCAGGTCCCAAACCC

Box II DR-a2 HTR 7-23 copies

CGAGCTCTGCCCAACTACTTTTCCTGCAAAAGGTCCCGACTAAAGAATCCTACCACAGACCCCCCCCCCCTTTTTTACCCGCTTACCCGC

TTACCCGCTTACCCGCTTACCCGCTTACCCGCTTACCCGCTTACCC

D) Silvery pout (*G. argenteus*)

DR-1 Box I

CCCTACTTATACCGGATACTGCCGTTAGGTCTACACTACTTCTGCCCTTG(C)$_{10-13}$ GCTC(A)$_{5-6}$ CCACCAGCGAACTCTACCACCTC

DR-2 Box II

TCCGTTATAAGAGAGGTNTTAATTCCCGCCCAACCCCTACTTCCCGTTTATACCAAAAACTGCCGTTAGATCTGCACTCGTTTCTACTTT

Box III

TTTCTCT(C)$_{10-11}$ AAAAAAGTTTAAATCAACTCCTCCCTATTCCGGAAACTGCCAGTGAACTCTACTACCTT
