# Supplementary material for: An evolutionary preserved intergenic spacer in gadiform mitogenomes generates a long noncoding RNA
Source: BMC Evol Biol. 2014 Aug 22;14:182. doi: 10.1186/s12862-014-0182-3 (PMC4236577; doi:10.1186/s12862-014-0182-3)
Supplement: Additional file 6: Figure S5. — Gadiform T-P spacer sequences. Box-motif is underlined. The different gadiform families are shown in A to G. [file s12862-014-0182-3-S6.pdf]

# Additional file 6: Figure S5

## A) Family: Gadidae

*Gadus morhua* (Atlantic cod); 74 bp; AM489716:
ACATCTAATTATTACCGGAGGACGCCCCATAATTCATCATCCACCGGAGGCTGCCCCATGGATCAAAATACCAC

*Gadus ogac* (Greenland cod); 73 bp; FJ396453:
ACATCTAATATTACCGGGTGACGCCCCATATATCATCATCTACCGGAGGCTGCCCCAGAGATCAAAATACCAC

*Theragra chalcogramma* (Alaska Pollock); 71 bp; Y17984:
ACATCTAACTATTACCGGATGACGCCCCATAATTATCAGCTACCGGAGCTGCCCCATGGTAAAAATACCAC

*Theragra finnmarchica* (Norwegian Pollock); 72 bp; AM489718:
ACATCTAACTATTACCGGATGACGCCCCATAATTATCAGCTACCGGAGGCTGCCCCATGGTAAAAATACCAC

*Boreogadus saida* (Polar cod); 70 bp; Y17985:
GCATCCAATTATTACCGGATGTAGCCCCATTTATATTAACCGGTAGCTGCCCCATGGATCAAAATACCGC

*Arctogadus glacialis* (Arctic cod); 291 bp; Ref [25]:
AGCATCTAATTATTACCGGGTGTAGCCCCACAATAAATAATAAATAATAAATAAATAAATAATAAATAAATAATAAATAA
ATAATAAATAAATAATAAATAAATAATAAATAAATAATAAATAAATAATAAATAAATAATAAATAAATAATAAATAAATA
ATAAATAAATAATAAATAAATAATAAATAAATAATAAATAAATAATAAATAAATAATAAATAAATAATAAATAAATAAAA
TTTATTTATTTATTTTATTTACCGGTAGCCGCCCCATGGTCAAAATACCAC

*Melanogrammus aeglefinus* (Haddock); 99 bp; Y17986:
GCAGCACACTACCGAAGCCTCATTATTTTCAACCGGTAACCGCCCCCCCATTAACATCAGCAACCATATTCTCAACCGGT
AGCCGCCCCACCAATAACA

*Merlangius merlangius* (Whiting); 69 bp; DQ020496:
ACAGCTTCTTATACCGGTTGCCGCCACATAATATTCTATGCCGGAAGCTGCCCTATATAACCTTCATAC

*Pollachius virens* (Saithe); 59 bp; FR751399:
TTTCGCGCGCGAATATTCTACTATCACCGGATGCTGCCCCATAATGTCGAAACATGTCT

*Pollachius pollachius* (Pollack); 50 bp; FR751400:
CAGCACGTCTTACTTTACCGGATGCTGCCCCATAATGTCGAAACACGTTC

*Trisopterus esmarkii* (Norwegian pout); 77 bp; This work:
TCCACTTTATCCACCCAACCTGCAGATGCAAAGACTACCGGATACTGCCCCAGGATACCCCGATCGACCAAACTTTT

*Trisopterus minutes* (Poor cod); 75 bp; This work:
CCGCCTTAGTAAATAGCTCTGCATAAGAAAGACTACCGGAAACTGCCCCATAACACCCAAATCGACCAAACTTTT

*Micromesistius poutassou* (Blue whiting); 48 bp; FR751401:
ACGCGCATATGCTCCGGGCACTGCCCCATAATGCTAAAACATTCCTGT

*Gadiculus argenteus* (Silvery pout); 258 nt; This work:
CCCTACTTATACCGGATACTGCCGTTAGGTCTACACTACTTCTGCCCTTGCCCCCCCCCCGCTCAAAAACCACCAGCGAA
CTCTACCACCTCTCCGTTATAAGAGAGGTNTTAATTCCCGCCCAACCCCTACTTCCCGTTTATACCAAAAACTGCCGTTA
GATCTGCACTCGTTTCTACTTTTTTCTCTCCCCCCCCCCAAAAAAGTTTAAATCAACTCCTCCCTATTCCGGAAACTGCC
AGTGAACTCTACTACCTT

## B) Family: Lotidae

*Lota lota* (Burbot); 103 bp; AP004412:
TATAGACACATAAATATTTAGTAAAGAGATTCAAAAATCTCTGATGCCACGAGCCGCGGCTCGTGGCTTTTCAACCGGAC
ACTGCCACCCCCCCCCCCCCAAC

*Brosme brosme* (Tusk); 48 bp; Y17988:
ACGCGCATATGCTCCGGGCACTGCCCCATAATGCTAAAACATTCCCTG

*Molva molva* (Ling); 83 bp; This work:
TCCAACATAAAGTGACTACCTCAGAGAGCTACTACACTTTCTCCAAACCGGGCTACGCCCCAAATAACCAATAAACGCCC
GCC

*Molva dipterygia* (Blue ling); 59 bp; This work:
CTAACAATAATTTTTCCCTTCGGGCCGGGCTACGCCCCAAATAGCCGAAACCGCCCGCC

## C) Family: Phycidae

*Enchelyopus cimbrius* (Fourbeaed rockling); 25 bp; Y17989:
CATTTACCGGTTTAGGCCCCACAAA

*Gaidropsarus argentatus* (Tree-bearded rockling); 48 bp; Y17990:
CTTTCCCGTGAGGCGCCTCACTAACCTCGTGGAGATGAGCCCGCACCC

*Phycis blennoides* (Greater forkbeard); 28 bp; This work:
TTTTACCCCGAGCTCTGCCTCACAATAC

## D) Family: Merlucciidae

*Macruronus novaezelandiae* (Hoki); 59 bp; This work:
TTTCGCGCGCGAATATTCTACTATCACCGGATGCTGCCCCATAATGTCGAAACATGTCT

*Merluccius merluccius* (European hake); 532 bp; FR751402:
AAACACGAGCAGCACAATTGCCTCCGAGCTCTGCCCAACCCACTTTCCTACAAAACCTTAAGAGTCTTAACCCCGCAGGT
CCCAAACCCCGAGCTCTGCCCAACTACTTTTCCTGCAAAAGGTCCCGACTAAAAAATCCTACCACAGACCCCCCCCCTTT
TTAAACCCGCCCTGCCCAGCCCACTTTCGTCAGGAAGACCCGGACCCAAAAAAATCTTCATCTTTCCCCCCCCCCCTTTT
TTTAACCCCCCCCCCCCCCCAAAGCCGGACCCAAAAAAATCTTCATCTTTCCCCCCCCCCCTTTTTTTAACCCCCCCCCA
AAGCCGGACCCAAAAAATCTTCATCCTTTTTTAACCCCCCCCTTTTTTACCCGCTTACCCGCTTACCCGCTTACCCGCTT
ACCCGCTTACCCGCTTACCCGCTTACCCGCTTACCCGCTTACCCGCTTACCCGCTTACCCGCTTACCCGCTTACCCGCTT
ACCCGCTTACCCGCTTACCCGCTTACCCGCTTACCCGCTTACCCGCTTACCC

## E) Family: Ranicipitidae

*Raniceps raninus* (Tadpole fish); 39 bp; This work:
ATCTCAACTTTTTATACCGGACACCGCCCCTTAACACAA

## F) Family: Macrouridae

*Squalogadus modificatus* (Tadpole whiptail); 47 bp; AP008989:
ACCTAGCACATTTTTTTACCGGGCTCTGCCCCAAATATTAAAATCAA

*Trachyrincus murragi* (Roughnose grenadier); 64 bp; AP008990:
TCACTTTTTTTCCAAGCTCTGCCCCAAATCAATGGAAATAAAGCTCAAAGGGGGAAGATTCGAA

*Bathygadus antrodes* (Anadara); 7 bp; AP008988:
ATATTTT

*Caelorinchus kishinouyei* (Mugara grenadier); 12 bp; AP002929:
CTAAACTACCCA

*Coryphaenoides rupestris* (Roundnose grenadier); 21 bp; This work:
CACCGACGTTCACCACCCACA

*Ventrifossa garmani* (Sagami grenadier); 72 bp; AP008991:
CTTCCCCACTCAGTTTTTTATTCCGAGTTCCGCCACACATATTTTAACGCAAAACGGCCCCCCGCCGGATGA

## G) Family: Moridae

*Physiculus japonicas* (Japanese codling); 16 bp; AP004409:
CACCGCCCTCTGCCAC

*Laemonema longipes* (Longfin codling); 33 bp; AB108839:
TCCCTTACCCCCGGACACCGCCACACTCTTCAA
