# Supplementary material for: An evolutionary preserved intergenic spacer in gadiform mitogenomes generates a long noncoding RNA
Source: BMC Evol Biol. 2014 Aug 22;14:182. doi: 10.1186/s12862-014-0182-3 (PMC4236577; doi:10.1186/s12862-014-0182-3)
Supplement: Additional file 7: Table S2. — Box-motif sequence compilation. [file s12862-014-0182-3-S7.pdf]

Additional file 7: Table S2

Box-motif sequence compilation

**Species**	**Box-motif** [1]	**Copy** [2]
**Gadiform codfishes**		
Gadus morhua	ACCGGAGGACGCCCCAT	I
Gadus ogac	ACCGGGTGACGCCCCAT	I
Theragra chalcogramma	ACCGGATGACGCCCCAT	I
Theragra finnmarchica	ACCGGATGACGCCCCAT	I
Boreogadus saida	ACCGGATGTAGCCCCAT	I
Arctogadus glacialis	ACCGGGTGTAGCCCCAC	I
Melanogrammus aeglefinus	ACCGGTAACCGCCCCCC	I
Merlangius merlangius	ACCGGTTGCCGCCACAT	I
Gadus morhua	ACCGGAGGCTGCCCCAT	II
Gadus ogac	ACCGGAGGCTGCCCCAG	II
Theragra chalcogramma	ACCGGA-GCTGCCCCAT	II
Theragra finnmarchica	ACCGGAGGCTGCCCCAT	II
Boreogadus saida	ACCGGTAGCTGCCCCAT	II
Arctogadus glacialis	ACCGGTAGCCGCCCCAT	II
Melanogrammus aeglefinus	ACCGGTAGCCGCCCCAC	II
Merlangius merlangius	GCCGGAAGCTGCCCTAT	II
Pollachius virens	ACCGGATGCTGCCCCAT	
Pollachius pollachius	ACCGGATGCTGCCCCAT	
Trisopterus esmarkii	ACCGGATACTGCCCCAG	
Trisopterus minutes	ACCGGAAACTGCCCCAT	
Micromesistius poutassou	TCCGGGCACTGCCCCAT	
Gadiculus argenteus	ACCGGATACTGCCGTTA	I
Gadiculus argenteus	ACCAAAAACTGCCGTTA	II
Gadiculus argenteus	TCCGGAAACTGCCAGTG	III
Lota lota	ACCGGACACTGCCACCC	
Brosme brosme	TCCGGGCACTGCCCCAT	
Molva molva	ACCGGGCTACGCCCCAA	
Molva dipterygia	GCCGGGCTACGCCCCAA	
Enchelyopus cimbrius	ACCGGTTTAGGCCCCAC	
Gaidropsarus argentatus	CCCGTGAGGCGCCTCAC	
Phycis blennoides	CCCGAGCTCTGCCTCAC	
Macruronus novaezelandiae	ACCGGATGCTGCCCCAT	
Merluccius merluccius	TCCGAGCTCTGCCCAAC	I
Merluccius merluccius	CCCGAGCTCTGCCCAAC	II
Raniceps raninus	ACCGGACACCGCCCCTT	
Squalogadus modificatus	ACCGGGCTCTGCCCCAA	
Trachyrincus murragi	TCCAAGCTCTGCCCCAA	
CONSENSUS	aCCggangctGCCccat	
**Ambystomatid salamanders**		
Ambystoma amblycephalum	ACCGGGCTATGC-TACG	
Ambystoma andersoni	ACCGGGCTATGCCTACG	
Ambystoma annulatum	ACCGGGCTATGC-TACG	
Ambystoma californiense	ACCAGGCTATGCCTACA	
Ambystoma cingulatum	ACCAGGCTCTGCTTACG	
Ambystoma dumerilii	ACCGGGCTATGCCTACG	
Ambystoma mexicanum	ACCGGGCTATGCCTACG	
Ambystoma opacum	ACCGGGCTATGC-TGCA	
Ambystoma talpoideum	ACCTAACTATGC-TGCA	
Ambystoma texanum	ACCAGGCAATGCCTACG	
Ambystoma tigrinum	ACCGGGCTATGCCTACG	
Ambystoma velasci	ACCGGGCTATGC-TACG	
CONSENSUS	ACCgggCtaTGCcTaCg	

Notes: [1] Box-motif sequences obtained from T-P spacers given in Additional file 6: Figure S5 (gadiform species) and references in the main text [17,18] (ambystomatid salamanders). Consensus sequences are presented for gadiform and salamander Box-motifs. Uppercase letters indicate 100% conserved nucleotide positions. Lowercase letters indicate majority nucleotide positions. 'n' indicates non-conserved nucleotide positions. Positions conserved between codfish and salamander are indicated by red letters. [2] Box-motif copy as given in Additional file 6: Figure S5.
