# Supplementary material for: An evolutionary preserved intergenic spacer in gadiform mitogenomes generates a long noncoding RNA
Source: BMC Evol Biol. 2014 Aug 22;14:182. doi: 10.1186/s12862-014-0182-3 (PMC4236577; doi:10.1186/s12862-014-0182-3)
Supplement: Additional file 8: Table S3. — PCR and sequencing primer specifications. [file s12862-014-0182-3-S8.pdf]

Additional file 8: Table S3

PCR and sequencing primer specifications

Primer [1]	Sequence (5´→3´)	Gene region
(T-P spacer PCR and sequencing primers):		
L15498	GAAACTGCCCTAGTAGCCA	tRNA-Thr
H15666	GTTTAATTTAGAATTCTAGCTTTGG	tRNA-Pro
(European hake PCR and sequencing primers):		
H112	CGGAGACTTGCATGTGT	SSU
L347	ACGAGCCCAAGTTGATA	SSU
H617	TAGAACAGGCTCCTCTAG	SSU
L1223	CGCAAGGGAACGCTGAAA	LSU
L2538	AATGGTGCAGCCGCTAT	LSU
L2776	TGTGTTAGAGTGGCAGAGC	tRNA-Leu1
H2812	CTCTGTAGAAAGGGCTTAGG	tRNA-Leu1
H3454	CTCTCCTTCAGTCAGGTCGA	ND1
L3760	TGGCACTAGTGATTTGACAT	ND1
L5072	GATAAACTAGACCAAGGGCCT	tRNA-Trp
H5481	GTGCCAATGTCTTTGAGATT	COI
L5862	CTTGCAAGTAATCTTGCCC	COI
L6842	CTAATCTCACTTCTAGCAAGT	COI
H7079	GGCAGTGTGATATTTCACCAG	tRNA-Asp
L7208	ATCACCCGTAATAGAAGAGT	COII
L7849	ACTTGAAGACGCCTCACTAAG	tRNA-Lys
L8016	GATAAACTAGACCAAGGGCCT	ATP8
H8073	CTTAGTGTCATGGTCAGTTTC	ATP8
L8864	CCTGCCGTATGGTTCCA	COIII
L9547	CAATCTATTGATGAGGCTC	tRNA-Gly
H10980	CCAGAGGTGTATTCCGTAGA	ND4
L11759	CTCTAATCCACCGAGAGAGGC	tRNA-His
H11887	ACTTGGAGTTGCACCAAGAG	tRNA-Leu2
L12809	CTGTGCCTCTGCCTCGGCG	ND5
H13950	TTGGGCCTCAGTTGAGGAGG	ND6
L14626	ACGGATGACTAATCCGCAAC	Cyt-B
H14746	AATTACGGTAGCTCCTCAGAATGATATTTGTCCTCA	Cyt-B
L15491	CTTGAATGAAACTGCCCTAG	tRNA-Thr
H15642	AGTTAGTGATGGGAGTTTAAGTC	tRNA-Pro
H16000	GGGTTGACAGGTTAAATACG	CR

Note: [1] The primers are numbered according to the 3’ nucleotide of the complete Atlantic cod mitogeneome sequence [21].
